# Supplementary material for: C/EBPβ Regulates TFAM Expression, Mitochondrial Function and Autophagy in Cellular Models of Parkinson’s Disease
Source: Int J Mol Sci. 2023 Jan 11;24(2):1459. doi: 10.3390/ijms24021459 (PMC9865173; doi:10.3390/ijms24021459)
Supplement: Supplementary file 1 [file ijms-24-01459-s001.zip › ijms-2094386-supplementary.pdf]

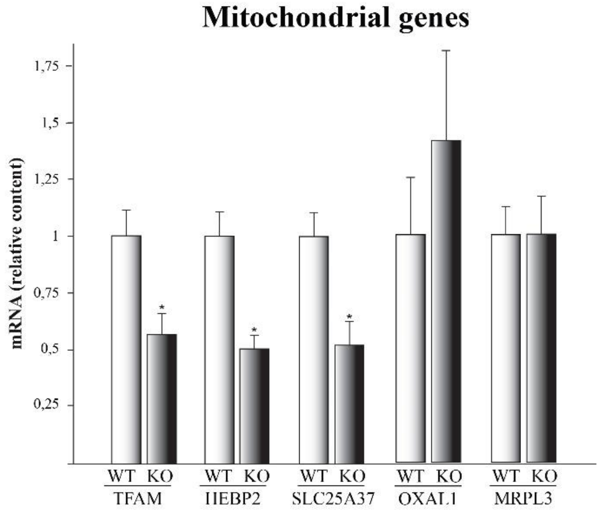

Supplementary Figure S1. Mitochondrial candidate genes confirmation by RT-qPCR in C/EBPβ-/- mice brain. RT-qPCR analysis of C/EBPβ+/+ (WT) and C/EBPβ-/- (KO) mice hippocampus mRNA of mitochondrial genes *Tfam*, *Hebp2*, *Slc25a37*, *Oxal1* and *Mrpl3*. Data are shown as the mean ± SE of the RNA relative levels in n=10 WT animals and n=5 KO animals. *p≤ 0.05 (t-student).

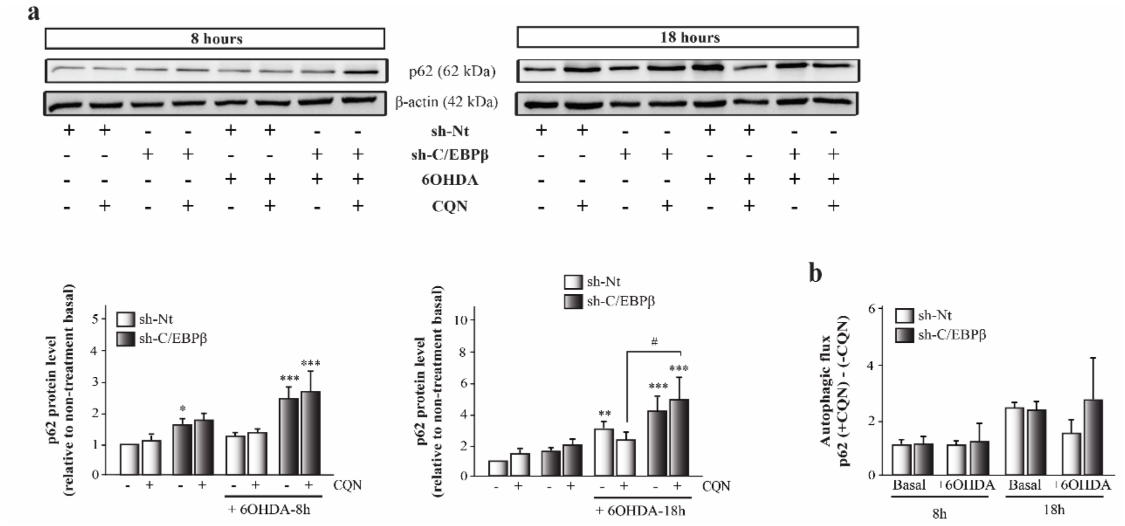

Supplementary Figure S2. Accumulation of autophagy markers in C/EBPβ silenced cells in PD models is due to the alteration of autophagosomes degradation. (**a**) Autophagy was inhibited with chlorquine (CQN, 30μM) in SH-SY5Y cells previously infected with lentiviral particles containing a control sh-Nt or with a shRNA targeting C/EBPβ (sh-C/EBPβ). Cultures were then treated with 35μM 6OHDA for 8 and 18 hours and immunoblot analyses were performed to determine p62/SQSTM protein levels. Data on the graphs correspond to the mean of the relative levels of p62 normalized by β-actin in at least 3 independent experiments ± SE. (**b**) Autophagic flux was calculated in by subtracting the value of p62 protein levels in the presence of CQN (+CQN) minus the value of this levels in the absence of CQN (-CQN) + 2 (to avoid negative values) in each experimental group. * p≤0.05, ** p≤0.005, *** p≤0.001 *vs* non-treatment basal, # p≤0.05 *vs* sh-Nt with the same treatments (ANOVA-Bonferroni).
